# Changes in Metabolites and Microbial Communities in Follicular Fluid Associated With Ovarian Function in Patients With Polycystic Ovary Syndrome

**DOI:** 10.1002/mco2.70622

**Published:** 2026-01-29

**Authors:** Manfei Si, Sen Yan, Shu Ding, Rui Liu, Xianglei Xiong, Jie Qiao, Xinyu Qi

**Affiliations:** ^1^ State Key Laboratory of Female Fertility Promotion, Center for Reproductive Medicine, Department of Obstetrics and Gynecology Peking University Third Hospital Beijing China; ^2^ National Clinical Research Center for Obstetrics and Gynecology (Peking University Third Hospital) Beijing China; ^3^ Key Laboratory of Assisted Reproduction (Peking University), Ministry of Education Beijing China; ^4^ Beijing Key Laboratory of Reproductive Endocrinology and Assisted Reproductive Technology Beijing China; ^5^ Institute of Advanced Clinical Medicine Peking University Beijing China

**Keywords:** follicular fluid, metabolite, microbiota, microecology, polycystic ovary syndrome

## Abstract

Polycystic ovary syndrome (PCOS) is a well‐documented endocrine disorder associated with metabolic abnormalities. Research has indicated potential links between PCOS and the gut microbiome, and the presence of microbial communities in follicular fluid (FF) has been demonstrated; however, their functional interplay with metabolites has not been elucidated. This case–control study involved 40 patients with PCOS and 40 controls matched for age. A comprehensive analysis of FF metabolites and microbial communities by means of metabolomics analysis and 16S rDNA sequencing was performed. Twelve metabolites and 15 microbial communities were significantly different between the PCOS and control groups. AMH and AFC were significantly associated with the majority of the differentially abundant metabolites and bacteria, suggesting a potential association between FF components and ovarian function. In this study, we found that D‐glucose and *Alicyclobacillus* were the most important variables in the metabolite model and microbial model, respectively. Mechanistically, *Alicyclobacillus acidoterrestris*, *Terrimonas ferruginea*, or *Terrimonas pekingense* can efficiently utilize glucose thereby reducing FF glucose levels, which provides insights into the microbiome–metabolite connection. These findings suggest a potential link among bacteria–metabolite–ovarian function, which could have implications for understanding the pathophysiology of PCOS and developing novel diagnostic and therapeutic strategies targeting metabolic and microbial aspects.

## Graphical abstract

Based on the integrated analysis of follicular fluid metabolites and microbial communities in patients with PCOS, this study reveals significant dysregulation in the follicular microenvironment. Key metabolites including D‐glucose and 25‐hydroxycholesterol show marked alterations, while microbial communities such as *Alicyclobacillus* and *Terrimonas* demonstrate distinctive abundance patterns. Importantly, specific bacteria like *Alicyclobacillus acidoterrestris* and *Terrimonas* species exhibit glucose‐utilizing capabilities that directly impact follicular fluid glucose levels. These findings establish a functional bacteria–metabolite–ovarian axis, where microbial metabolic activities interact with host metabolites to influence ovarian function, providing new insights into PCOS pathophysiology and potential therapeutic targets.

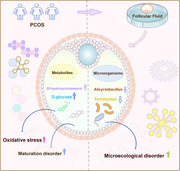

## 1 Introduction

Polycystic ovary syndrome (PCOS) represents the leading reproductive endocrine disorder among women of childbearing years, with a global prevalence approximately 10%–13% [1]. The main clinical manifestations of PCOS are clinical or biochemical hyperandrogenemia, with manifestations including acne, hirsutism and alopecia, polycystic ovarian morphology, menstrual disorders, and sparse/no ovulation, resulting in 80% of anovulatory infertility [2]. Although women with PCOS are more likely to have a successful pregnancy with the help of assisted reproductive technology (ART), relative to those unaffected individuals, women diagnosed with PCOS are at a significantly higher risk of adverse obstetric outcomes including miscarriage and preterm delivery [3, 4, 5]. These findings suggest that oocyte quality may be impaired in patients with PCOS.

Follicle fluid (FF) is a complex biological fluid microenvironment found within follicles that plays a crucial regulatory and supportive roles in the growth, maturation, and developmental potential of oocytes [6]. FF comes from follicular cell secretions and plasma components and is rich in hormones, growth factors, nutrients, and metabolites [7]. This unique microenvironment not only provides necessary nutritional substrates and energy sources for oocytes but also serves as a medium for signal molecule transmission, mediating bidirectional communication between oocytes and surrounding granulosa cells [8]. Dynamic changes in the composition of follicular fluid directly affect the nuclear and cytoplasmic maturation of oocytes, thereby determining their fertilization ability and potential for subsequent embryonic development [9]. Therefore, the composition and quality of follicular fluid can be used for evaluating the health and developmental ability of oocytes and have significant implications for ART.

A pronounced proinflammatory environment is present in the FF of patients with PCOS, resulting in elevated generation of reactive oxygen species (ROS), accumulation of lipid peroxidation byproducts, and compromised antioxidant defense mechanisms [10, 11, 12, 13, 14]. Multiple proteomic analyses of FF from patients with PCOS have revealed significant alterations in pathways associated with the immune response, metabolic processes, angiogenesis, and hormone regulation [15, 16, 17, 18]. In particular, changes in the FF metabolic profile may reflect underlying metabolic disorders in patients with PCOS and may offer further understanding on the metabolic interplay involving granulosa cells and oocytes. Metabolomic analysis plays a pivotal role in understanding and managing human diseases by providing a dynamic readout of pathological states [19]. Multiple metabolomic studies have revealed disturbances in glucose metabolism, amino acid levels, lipid profiles, and steroid levels in the FF of patients with PCOS [20, 21, 22, 23]. Changes in these metabolites may disrupt the immune homeostasis of FF and adversely affect oocyte development in patients with PCOS. These results emphasize the important role of the metabolic characteristics of FF in oocyte development.

In addition to active substances, recent studies have highlighted the presence and significance of microorganisms in FF [24, 25]. The abundance of these microorganisms is low, but their physiological functions are still of concern as they may directly regulate oocyte development by producing certain active metabolites [22]. However, whether microorganisms in FF have adverse effects on assisted reproductive outcomes remains controversial [24, 25, 26, 27, 28, 29]. Pelzer et al. reported that the colonization of FF by microorganisms was associated with reduced fertilization rates in women with PCOS, but it is still unclear whether this difference is related to decreased reproductive ability, and the key mediators involved have yet to be identified [30]. Furthermore, the full picture of the microbial signature of FF in PCOS patients is not yet fully understood, and the relationship between microorganisms and metabolites has not been resolved.

Limited research has been conducted on this topic, and how the metabolic and microbial characteristics of FF affect ovarian function and assisted reproductive outcomes in patients with PCOS remains unclear. These knowledge gaps hinder our understanding of the microbial functions of FF. Elucidating changes in FF in women with PCOS is key to understanding the mechanism underlying the low fertility in this population. Therefore, the aim of this study was to focus on and elucidate the bacterial composition and metabolite profile in FF obtained from patients with PCOS receiving ART treatment and to determine its potential value for predicting pregnancy outcomes. In this study, the goal was to identify key microorganisms or metabolites directly related to the occurrence of PCOS by screening candidate targets for the clinical management of PCOS and providing a solid theoretical foundation for the development of clinical diagnostic and treatment strategies.

## 2 Results

### 2.1 General Characteristics of the Patients and Clinical Pregnancy Outcomes

As shown in Table 1, the PCOS and control groups showed no significant differences in age, body mass index (BMI), basal follicle‐stimulating hormone (FSH) levels, estradiol (E_2_) levels or metabolic indices such as triglyceride (TG), total cholesterol (TC), high‐density lipoprotein (HDL‐C), low‐density lipoprotein (LDL‐C), and fasting blood glucose levels. In contrast, the PCOS group exhibited a prolonged duration of infertility. Significant disparities were observed in serum luteinizing hormone (LH), testosterone (T), androstenedione (AND), anti‐Müllerian hormone (AMH), and antral follicle count (AFC) levels, findings that align with the typical clinical presentation of PCOS. The influence of metabolic diseases on the detection of metabolites in FF was excluded. In the PCOS group, both the initial FSH dosage and the total dosage of gonadotropin were considerably lower than those in the control group (*p* < 0.001). Compared with that in the control group, the rate of frozen embryo transfer in the PCOS group was significantly greater (*p* < 0.001). The normal fertilization, cleavage, high‐quality embryo, available embryo, first and cumulative clinical pregnancy rates, first and cumulative live birth rates, first and cumulative pregnancy complication rates, first and cumulative preterm birth rates, and first and cumulative low birth weight infant rates did not differ significantly between the PCOS group and the control group (Table S1).

**TABLE 1:** General patient characteristics in the PCOS and control groups.

Variable	PCOS (*n* = 40)	Control (*n* = 40)	*p* value
Age (year)	31.10 (3.39)	31.62 (3.36)	0.465
BMI (kg/m^2^)	23.16 (1.94)	22.63 (2.36)	0.184
Duration of infertility (years)*	4.00 (3.75)	2.50 (4.00)	0.040
Infertility diagnosis			
Primary infertility	29 (72.5%)	31 (77.5%)	0.606
Secondary infertility	11 (27.5%)	9 (22.5%)	
Basal FSH (mIU/mL)*	5.98 (1.72)	6.33 (2.77)	0.386
Basal LH (IU/L)*	5.05 (2.42)	2.68 (1.60)	4.160E−7
Basal E_2_ (pmol/L)*	123.50 (70.5)	112.50 (50.00)	0.308
Basal P (ng/mL)	0.95 (0.35)	1.02 (0.44)	0.394
Basal T (nmol/L)*	1.02 (0.57)	0.69 (0.00)	4.000E−6
Basal AND (nmol/L)*	10.45 (5.23)	5.29 (3.45)	2.903E−7
AMH (ng/mL)*	8.37 (4.90)	2.93 (2.52)	1.070E−10
Bilateral AFCs*	24.00 (6.00)	15.50 (8.00)	5.162E−13
TG (mmol/L)*	0.96 (0.49)	0.78 (0.54)	0.197
TC (mmol/L)	4.52 (0.89)	4.45 (0.84)	0.724
HDL‐C (mmol/L)*	1.28 (0.30)	1.38 (0.46)	0.207
LDL‐C (mmol/L)	2.60 (0.67)	2.50 (0.60)	0.467
Fasting blood glucose (mmol/L)	4.96 (0.46)	5.06 (0.49)	0.371
Initial FSH dosage (IU/day)*	150.00 (21.90)	168.75 (93.80)	9.300E−5
Duration of stimulation (days)*	10.00 (2.00)	10.00 (3.00)	0.123
Total dose of gonadotropin (IU)*	1418.75 (681.30)	2268.75 (1256.30)	2.100E−5
Endometrial thickness (mm)*	10.00 (3.00)	11.00 (2.00)	0.205
Number of retrieved oocytes*	17.50 (11.00)	12.00 (8.00)	4.080E−4
Insemination method			
IVF	24 (60.0%)	10 (25.0%)	0.003
ICSI	16 (40.0%)	27 (67.5%)	
Half‐ICSI	0 (0.0%)	3 (0.75%)	

*Note*: Continuous data were reported as the mean (standard deviation) for normally distributed data or the median (interquartile range) for non‐normally distributed data*. Categorical data were reported as *n* (%). The *t*‐test or Mann–Whitney *U*‐test was used to analyze continuous data, and the chi‐square test or Fisher's exact test was used to analyze categorical data.

Abbreviations: AFC, antral follicle count; AMH, anti‐Müllerian hormone; AND, androstenedione; BMI, body mass index; E2, estradiol; FSH, follicle‐stimulating hormone; HDL‐C, high‐density lipoprotein; ICSI, intracytoplasmic sperm injection; IVF, in vitro fertilization; LDL‐C, low‐density lipoprotein; LH, luteinizing hormone; P, progesterone; T, testosterone; TC, total cholesterol; TG, triglyceride.

### 2.2 Characteristics of Metabolites in the Follicular Fluid of Patients With PCOS

**FIGURE 1:**
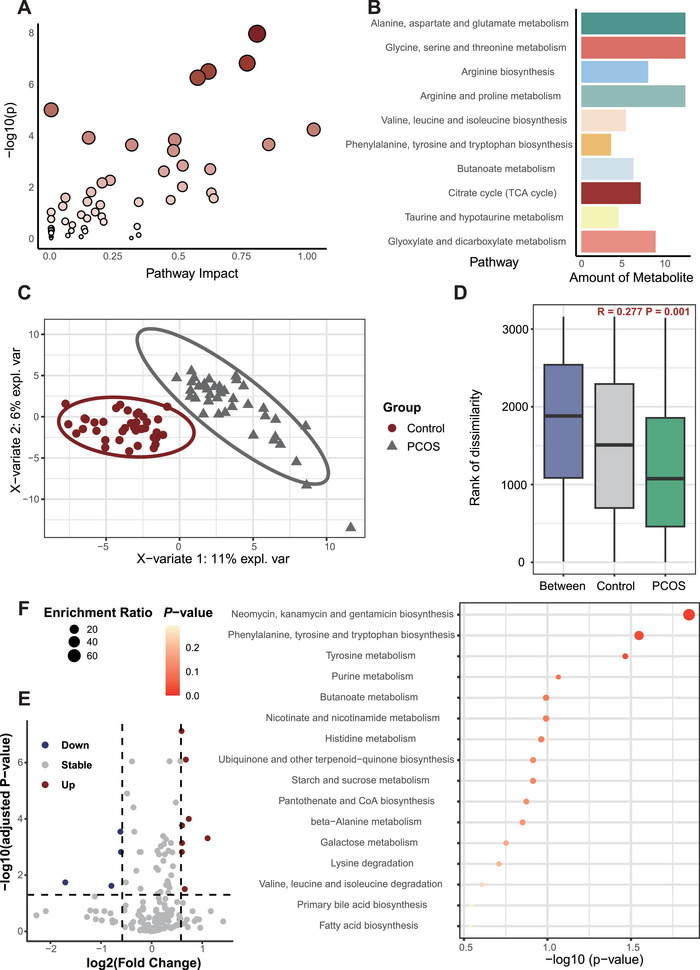
Extensive changes in the metabolic profile of follicular fluid associated with patients with PCOS. (A) Pathway distribution of all the metabolites. (B) The top 10 metabolic pathways associated with all the metabolites. (C) Partial least squares discriminant analysis of different groups of metabolites. (D) Similarity analysis of metabolite distribution (Anosim) in different groups. (E) Volcanic distribution map of different metabolites in the PCOS group and control group. (F) KEGG analysis of differentially abundant metabolites.

We conducted a metabolomic analysis of FF from different population groups. Following the exclusion of metabolites whose concentrations were too low for precise identification, a total of 182 metabolites were annotated. These metabolites were involved in 53 metabolic pathways, with amino acid metabolic pathways accounting for 28.3% of these pathways (Figure 1A,B). The results of the principal component analysis (PCA) revealed discernible disparities in metabolite distribution between the control and PCOS groups (Figure S1A). Subsequent partial least squares discriminant analysis (PLS‐DA) revealed clear segregation of the PCOS group from the control group into two distinct clusters (Figure 1C). Furthermore, similarity analysis of metabolites (Anosim) confirmed significant differences between the two groups (*p* = 0.001; Figure 1D). *P* values and FC values were subsequently used to identify the characteristic metabolites responsible for the observed differences. As present in Figure 1E, compared to the control group, 12 metabolites differed in the PCOS group. In particular, eight metabolites were upregulated (25‐hydroxycholesterol, D‐glucose, 4‐hydroxyphenylpyruvic acid, pantothenic acid, acetoacetic acid, allantoin, xanthine, and malonic acid), and four were downregulated (O‐phosphotyrosine, quinolinic acid, 4‐trimethylammoniobutanoic acid, and histidine). These differentially abundant metabolites belong to different metabolic pathways (Figure 1F).

### 2.3 Characteristics of Microorganisms in the Follicular Fluid of Patients With PCOS

**FIGURE 2:**
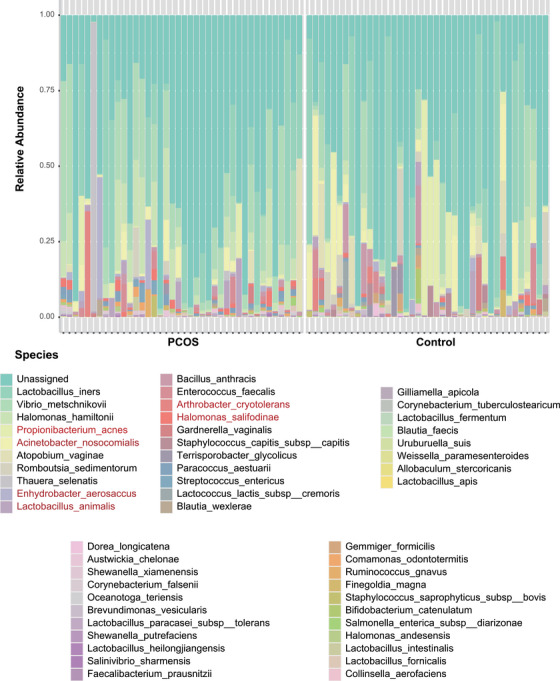
The relative abundance of different species in the PCOS group and the control group. The relative abundance of each species was indicated by different colors.

**FIGURE 3:**
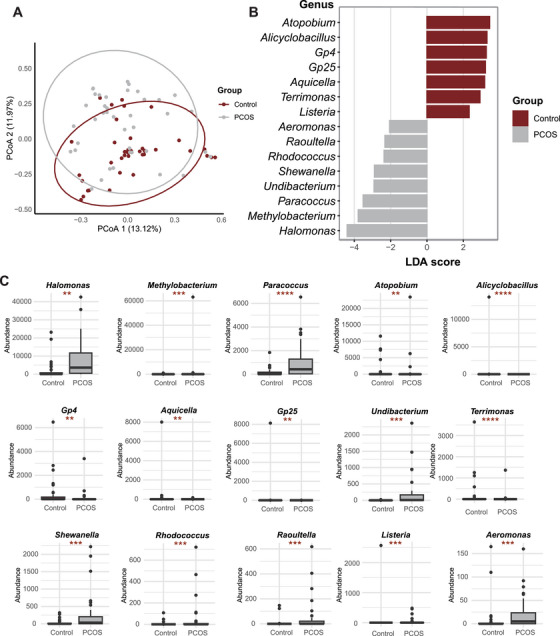
Extensive changes in the microorganisms of follicular fluid associated with patients with PCOS. (A) β‐Diversity analysis using principal coordinate analysis. (B) LEfSe analysis of the microbiota community between groups with linear discriminant analysis scores. (C) Distribution of microbial abundance for the 15 differential genera.

As shown in Figure 2, compared with that in the control group, the composition of several microbial species significantly changed in the PCOS group. In particular, the differences in *Propionibacterium_acnes*, *Acinetobacter_nosocomialis*, *Enhydrobacter_aerosaccus*, *Lactobacillus_animalis*, *Arthrobacter_cryotolerans*, and *Halomonas_salifodinae* were most obvious. The microbial communities between the two groups overlapped to some extent, but the distribution in the PCOS group was more dispersed (Figure 3A). As seen from the alpha diversity analysis (Figure S1B), both the Shannon index and Simpson index of the PCOS group were lower than those of the control group, indicating that the species diversity of the PCOS group had decreased. However, among the eight indices, only the goods coverage index significantly differed.

On this basis, we used LEfSe (Figure 3B) to analyze the disparities in the FF microbiota between the PCOS and control groups. As shown in Figure 3B, 15 different microorganisms were identified in the PCOS group compared with the control group, with eight genera (*Aeromonas*, *Raoultella*, *Rhodococcus*, *Shewanella*, *Undibacterium*, *Paracoccus*, *Methylobacterium*, and *Halomonas*) increasing in abundance and seven genera (*Listeria*, *Terrimonas*, *Aquicella*, *Gp25*, *Gp4*, *Alicyclobacillus*, and *Atopobium*) decreasing in abundance. We subsequently quantified the abundance of these genera (Figure 3C), revealing significant upregulation or downregulation in PCOS patients. These findings provide further evidence for the correlation between these microorganisms and the occurrence of PCOS. However, a direct association with PCOS has not been previously documented.

### 2.4 Correlation Analysis of Follicular Fluid Metabolites, the Microbiome and Clinical Indicators

**FIGURE 4:**
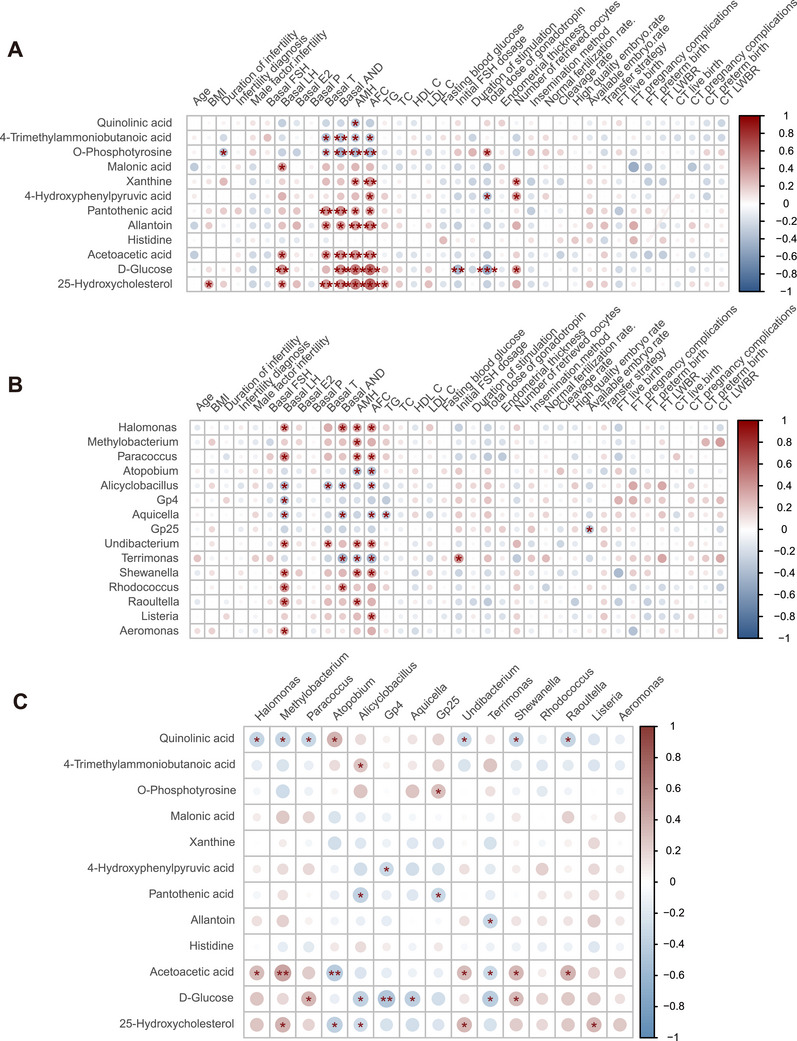
Correlation analysis between clinical features and the follicular fluid microenvironment in patients with PCOS. (A) Spearman correlation analysis of clinical features and differential metabolites. (B) Spearman correlation analysis of clinical features and differential microorganisms. (C) Spearman correlation analysis of differential metabolites and differential microorganisms.

There were significant differences in metabolic profiles and microbial diversity between the PCOS and control groups, with 12 distinct metabolites and 15 differential microbial communities identified. Spearman's correlation analysis was performed to investigate associations between clinical indicators, FF metabolites and FF microbial community composition. Notably, T and AND were correlated with more than 50% of the differentially abundant metabolites (Figure 4A). In addition, both AMH and AFC were significantly associated with the majority of the differentially abundant metabolites, demonstrating the potential regulatory role of FF metabolites in ovarian reserve function (Figure 4A). As shown in Figure 4B, LH, AMH, and AFC were linked to most of the differential bacteria. Specifically, T and AND were negatively correlated with *Alicyclobacillus*, whereas AND was significantly negatively correlated with *Aquicella* and *Terrimonas*. These findings indicate a possible link between the components of FF and ovarian function, although the underlying biological mechanisms remain to be further investigated.

Moreover, a robust correlation was observed between microbial diversity and metabolite profiles in FF (Figure 4C). In total, 31 metabolite–microbial community associations were identified, 13 of which were positively correlated, 18 of which were negatively correlated, and three of which were significantly correlated (*p* < 0.01). Specifically, the acetoacetic acid level was positively correlated with *Methylobacterium* and negatively correlated with *Atopobium*. Additionally, a significant negative correlation was observed between D‐glucose levels and the abundance of *Gp4*. These findings collectively suggest the existence of intricate, yet uncharacterized, crosstalk among microbial communities, metabolic signatures, and clinical phenotypes. Future research is required to explore the potential mechanisms driving these interactions.

### 2.5 Prediction of PCOS With Multiomics Features in Follicular Fluid

**FIGURE 5:**
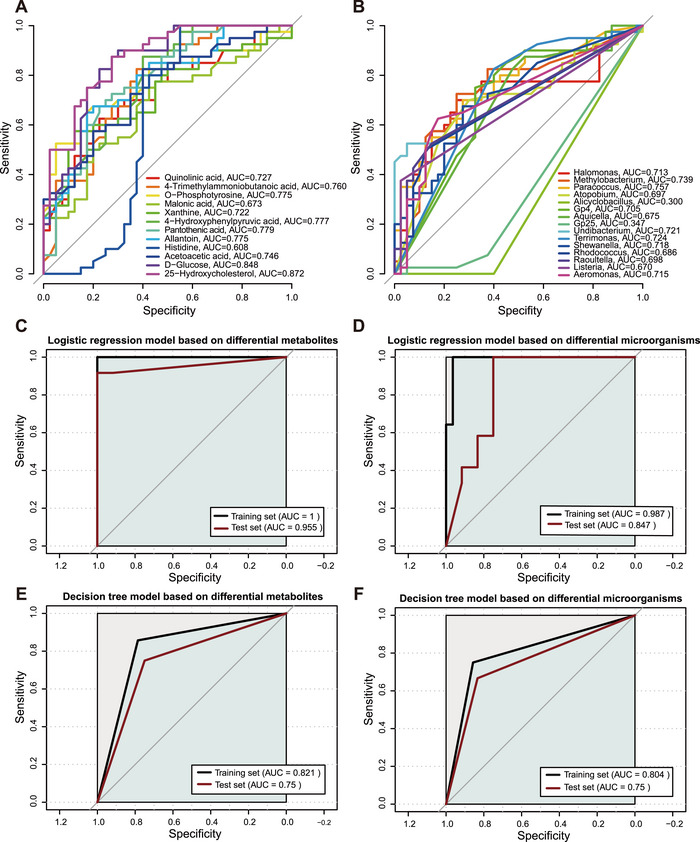
The predictive performance of differential metabolites and differential microorganisms for PCOS. (A) ROC of the differentially abundant metabolites. (B) ROC of the differential microorganisms. (C) Logistic regression model based on differential metabolites. (D) Logistic regression model based on differential microorganisms. (E) Decision tree model based on differential metabolites. (F) Decision tree model based on differential microorganisms. ROC, receiver operating characteristic curve.

As shown in Figure 5A, we performed a receiver operating characteristic curve (ROC) analysis to evaluate the diagnostic potential of the differentially abundant metabolites. All but one of the differentially abundant metabolites exhibited good diagnostic performance (area under the curve [AUC] > 0.65). Similarly, when using the differentially abundant bacteria to assess diagnostic performance, two of the bacterial markers demonstrated poor diagnostic efficacy (Figure 5B).

**FIGURE 6:**
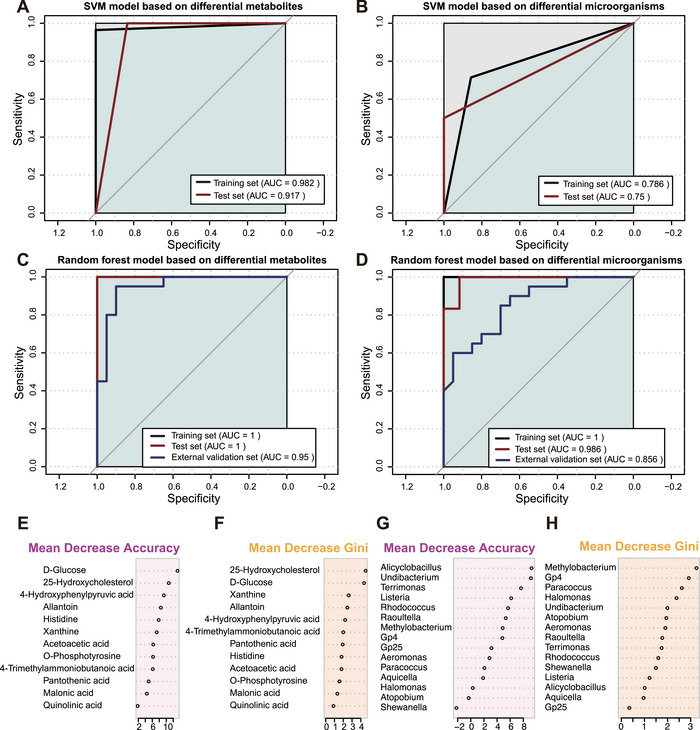
Evaluate the classification performance of different machine learning models based on the ROC. (A) SVM model based on differential metabolites. (B) SVM model based on differential microorganisms. (C) Random forest model based on differential metabolites. (D) Random forest model based on differential microorganisms. (E, F) The mean decrease accuracy and mean decrease Gini of each differential metabolite in the random forest model. (G, H) The mean decrease accuracy and mean decrease Gini of each differential microorganism in the random forest model. ROC, receiver operating characteristic curve; SVM, support vector machine.

We also developed a multivariable machine learning prediction framework, which adopts four algorithms: binary logistic regression (BLR), decision tree (DT), random forest (RF), and support vector machine (SVM; Figures 5C–F and 6A–D). It was used to assess the comprehensive predictive ability of differentially abundant metabolites and microorganisms. Among these four models, the RF model had the best predictive performance for both the metabolites (AUC = 1 for the training set and AUC = 1 for the test set; Figure 6C) and the microbiota (AUC = 1 for the training set and AUC = 0.986 for the test set; Figure 6D). Compared with that of the single‐index models, the performance of the multi‐index model significantly improved. As the RF model had the best predictive performance, we further verified its predictive accuracy in an external validation set (*n* = 20 PCOS patients vs. *n* = 20 controls). This model also demonstrated excellent predictive ability in the external validation set (AUC = 0.950 for the metabolites and AUC = 0.856 for the microorganisms; Figure 6C,D). We calculated the mean decrease in accuracy and mean decrease in the Gini coefficient of each marker in the RF model. D‐glucose and 25‐hydroxycholesterol were the most important variables in the metabolite model (Figure 6E,F). *Alicyclobacillus* and *Methylobacterium* were the most important variables in the microbial model (Figure 6G,H).

The existing diagnostic criteria for PCOS are already concise and clear enough. In daily clinical practice, incorporating multiomics analysis of FF into the routine diagnostic process is unnecessary. However, the findings of this study do indeed reveal significant differences in the composition of metabolites and microbiota in the FF of patients with PCOS. The results of the model further highlight the biological significance of the FF microecological environment in regulating ovarian function. These significantly different metabolites and microorganisms may provide new perspectives and strategies for research on the underlying mechanism and treatment of PCOS.

### 2.6 Mechanistic Insights Into the Association Between Microbes and Metabolites

**FIGURE 7:**
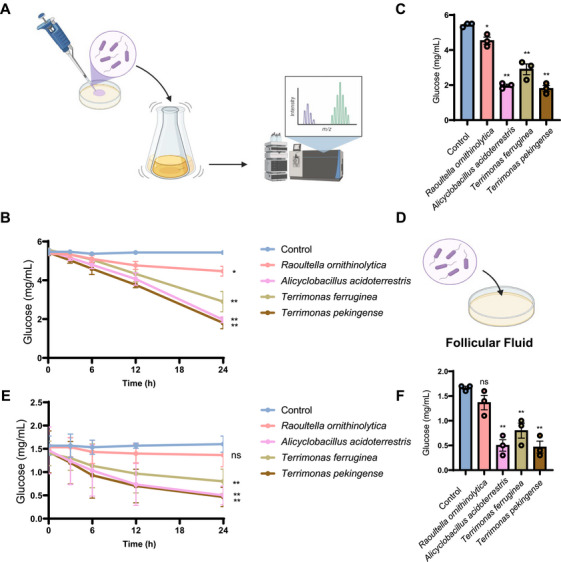
The interaction between microorganisms and metabolites in the follicular fluid. (A) Flow chart for detecting the glucose metabolism ability of different microorganisms in standard bacterial culture medium. The figure was created in BioRender (https://BioRender.com/rzpabgv). (B) Dynamic curves of glucose metabolism by different microorganisms in standard bacterial culture medium. (C) Remaining glucose content in the culture medium after incubation with microorganisms for 24 h. (D) Flow chart for detecting the glucose metabolism ability of different microorganisms in follicular fluid. The figure was created in BioRender (https://BioRender.com/rzpabgv). (E) Dynamic curves of glucose metabolism by different microorganisms in follicular fluid. (F) Remaining glucose content in follicular fluid after incubation with microorganisms for 24 h.

Based on the key metabolites and microbiota identified through the aforementioned RF models, we verified the potential microbial–metabolite associations in the follicular fluid. As shown in the Figure 6E,G, D‐glucose and *Alicyclobacillus* were the most important variables in the metabolite and microbial model, respectively. In terms of the significant correlation between D‐glucose and various bacteria in the follicular fluid, and obtained representative species (*Alicyclobacillus acidoterrestris*, *Terrimonas ferruginea*, and *Terrimonas pekingense*) from genus *Alicyclobacillus* and *Terrimonas*, each of which presented a distinct negative association with glucose. Use *Raoultella ornithinolytica* as a negative control because its poor correlation with glucose. We propagated them in standard bacterial culture medium, and tested their ability to metabolize glucose (Figure 7A). We monitored the glucose content in the culture medium and found that the addition of *Alicyclobacillus acidoterrestris*, *Terrimonas ferruginea*, or *Terrimonas pekingense* consumed the glucose in the culture medium rapidly (Figure 7B), indicating active glucose metabolism. Under the same conditions, glucose levels will not change in the culture medium without the addition of microorganisms (Figure 7C). Furthermore, to simulate a realistic environment in follicular fluid, we injected *Alicyclobacillus acidoterrestris*, *Terrimonas ferruginea*, or *Terrimonas pekingense* into the follicular fluid (Figure 7D) and found that the bacteria significantly reduced the glucose levels in the follicular fluid (Figure 7E,F). Compared with them, *Raoultella ornithinolytica* exhibits weak metabolic capacity for glucose. In summary, we found that *Alicyclobacillus acidoterrestris*, *Terrimonas ferruginea*, or *Terrimonas pekingense* can efficiently utilize glucose, thereby reducing follicular fluid glucose levels. This provides insights into the microbiome–metabolite connection and biologically confirms the significant negative correlation both *Alicyclobacillus* and *Terrimonas* with glucose that we observed in follicular fluid. In summary, these results further strengthen the biological significance of our conclusions and establish a reliable causality among bacteria–metabolites–ovarian function.

## 3 Discussion

In this study, we integrated metabolomics analysis with 16S rDNA sequencing to conduct a comprehensive assessment of the dynamic changes in the FF microecology of patients with PCOS. Importantly, this study revealed 12 distinct metabolites and 15 specific bacterial genera that were significantly associated with the development of PCOS in FF. To the best of our knowledge, there are no prior studies examining the correlation between FF metabolites and microbial communities in PCOS patients. The findings revealed that both the richness and diversity of metabolites and microbial communities in the FF of PCOS patients were altered. The dynamic changes in these bacterial genera within the FF of PCOS patients might regulate the levels of key metabolites related to PCOS, thus inducing abnormal follicular function.

As a noninvasive test, FF provides a window for evaluating the intrafollicular microenvironment, and identifying abnormalities in its composition may elucidate the mechanisms that lead to abnormal oocyte development [31]. Numerous recent studies highlight the critical role of FF metabolic profiling in PCOS pathophysiology [20, 32, 33, 34]. The widely targeted metabolomics we use provides a new perspective on key features in PCOS.

KEGG pathway analysis shows that amino acid metabolism and lipid metabolism in follicular fluid of PCOS patients are disturbed, which is a common feature of PCOS patients. Some key amino acids have been shown to be associated with the onset of PCOS. For example, arginine is widely known for its pro PCOS effect of its microbial metabolite agmatine, which inhibits host GLP‐1 secretion by activating FXR signaling, thereby inducing PCOS [35]. In addition, the metabolic pathways of aromatic amino acids (phenylalanine, tyrosine, and tryptophan) and branched chain amino acids (valine, leucine, and isoleucine) have been enriched in many related studies, which are related to lipid metabolism and linked together with microbial–host cometabolism [36, 37, 38]. Many aspects of these metabolites have not been fully elucidated, however, previous studies and our results suggest that these metabolites may play an important role in the pathogenesis of PCOS. Although many interesting associations have not been thoroughly clarified, we have listed some novel results that will be analyzed thoroughly in the subsequent text.

Elevated acetoacetic acid and D‐glucose levels in FF, as observed in this study, align with systemic insulin resistance and mitochondrial dysfunction in PCOS pathogenesis [33]. As reported in the literature, changes in FF metabolic profiles may reflect potential metabolic disorders in individuals with PCOS and subsequently affect oocyte quality and fertility [39, 40]. Trimethylammoniobutanoic acid is a precursor of L‐carnitine (LC), which plays important roles in fatty acid β‐oxidation and mitochondrial energy metabolism. Emerging evidence indicates that reduced LC levels may lead to abnormal metabolic processes in patients with PCOS, and multiple studies have confirmed the benefit of LC supplementation to improve insulin sensitivity and ovulation rates as a therapy for patients with PCOS [41, 42, 43]. Notably, our findings align with those of another study in which 4‐trimethylammoniobutanoic acid was significantly downregulated in the FF of patients with PCOS, and its diagnostic performance alone was surprisingly close to 0.777 [39].

Acetoacetic acid, a ketone body marker of impaired energy homeostasis, accumulates in individuals with PCOS through insulin resistance‐mediated pathways. Insulin deficiency or resistance triggers excessive adipose tissue lipolysis and then leads to elevated levels of acetoacetic acid [44]. Patients with PCOS often have insulin resistance, which promotes lipolysis and ketone body production [45]. Acetoacetic acid can induce oxidative stress [46, 47, 48]. Therefore, the increase in its level may be related to the oxidative stress state in the FF, which in turn affects oocyte quality and follicle development.

D‐glucose is an essential energy substrate for oocyte maturation, fertilization and embryonic development [49]. Glucose in FF provides metabolic support for follicle growth and development through aerobic oxidation in both oocytes and granulosa cells. Normally, granulosa cells convert glucose to lactate via glycolysis to supply energy for oocyte development [50]. Metabolic disorders in patients with PCOS may cause changes in glucose levels in FF. The characteristics of ovarian glucose metabolism in patients with PCOS are reduced glucose consumption in and uptake by oocytes, enhanced glycolysis in follicular fluid, and decreased glucose metabolism in granular cells.

Metabolic shifts correlate with altered microbial communities, and FF microecology thus represents a critical window for understanding PCOS‐related fertility challenges through integrated metabolite–microbe analyses. Our identification of 15 differential microbial taxa, including the marked depletion of *Lactobacillus* species and enrichment of *Propionibacterium acnes* in patients with PCOS, echoes findings from recent microbiome studies demonstrating altered reproductive tract microbiota in patients with PCOS [51, 52, 53]. Notably, the negative correlation between androgen levels (T and AND) and *Alicyclobacillus* abundance supports the hypothesis that hyperandrogenemia may create selective pressure that shapes the microbial ecology in FF, potentially through pH modulation or immune pathway activation [54, 55, 56]. These microbial shifts could disrupt follicular homeostasis, as evidenced by the significant associations between differential taxa (e.g., *Aquicella*) and ovarian reserve markers (AMH/AFC), which is consistent with the findings of animal models linking alterations in the gut microbiota to ovarian dysfunction [57]. Notably, the negative association between AND and *Terrimonas* introduces a novel microbial player in hyperandrogenism pathophysiology. This soil‐derived genus, rarely reported in human microbiomes, may be involved in steroid hormone metabolism through yet‐uncharacterized enzymatic activities, paralleling known microbial capacities for estrogen modification [58, 59, 60]. Although the association between androgens and microorganisms in FF has not been elucidated, we propose some interesting mechanisms. Some microorganisms are rich in steroid degrading enzymes, which are believed to have the ability to inactivate androgens [61, 62]. This function may be lacking in PCOS. On the other hand, high androgen levels in FF may regulate the growth of certain microorganisms, which may be related to the onset of PCOS. The example of this hormone regulating microbial growth has been widely studied previously [63, 64]. The positive correlation between D‐glucose and Gp4 (a poorly characterized *Firmicutes* genus) raises questions about microbial involvement in follicular glucose metabolism, a crucial factor in oocyte competence, given the established link between FF glucose levels and embryo quality [33, 65, 66].

Our findings also indicated a lack of significant association between the abovementioned ART outcomes and FF microecology. When our research results are analyzed, several key factors must be considered. First, the limitation of the sample size may have affected the effectiveness of the statistical analysis, which suggests that in future work, we need to recruit a larger sample population to increase the reliability and universality of the research results. Second, clinical pregnancy, live birth, pregnancy complications and preterm birth, and other pregnancy outcomes are complex processes that are determined by multiple factors. These outcomes may be influenced by multiple factors such as genetics, lifestyle, environmental factors, and medical interventions. A single microenvironmental indicator of follicular fluid may not be sufficient to fully explain the variations in these long‐term pregnancy endpoints. Besides, the physical condition of these patients had already been adjusted to optimal levels through lifestyle interventions or medication treatment before undergoing ART, thereby weakening the link between PCOS and ART outcomes. In addition, personalized IVF protocols have been developed by clinical doctors based on patient biochemical indicators, which can maximize the improvement of patient fertility outcomes. This may also be the reasons for the poor correlation between FF microbiota and ART outcomes. Given the significant importance of using FF to predict ART outcomes, this will continue to be our focus in future research. To achieve a deeper insight into the complex mechanisms influencing pregnancy outcomes, it is necessary to comprehensively consider multiple biomarkers and clinical factors.

Previous studies have demonstrated the presence of microbial communities in follicular fluid, but the functional interplay between these microbial communities and metabolites has not been explored [24, 67, 68]. For the first time, we discovered the presence of tissue‐specific microbes in FF that are correlated with follicular dysfunction and the development of PCOS. The source of the microbes in the follicular fluid is still controversial. The detection of distinct microbial communities in FF challenges the traditional view of the ovarian follicle as a sterile microenvironment. Our findings, combined with emerging evidence, suggest multiple potential routes for the microbial colonization of FF. The FF microbiota likely originates through external translocation (e.g., vaginal *Atopobium* ascension via cervical transfer) and iatrogenic introduction during oocyte retrieval (e.g., environmental *Terrimonas* contamination), despite sterile protocols. In our research, the nucleic acid‐free water was confirmed to contain no detectable microorganisms. Low‐biomass FF samples may be susceptible to technical artifacts. While our study employed stringent controls to minimize contamination, we cannot exclude the possibility that some microbial signals are derived from procedural artifacts or passive diffusion. Interestingly, we detected significant differences between the microbiota in FF and common vaginal and intestinal microbiota. In the intestine, it is mainly composed of *Firmicutes* and *Bacteroidetes* [69], whereas in the vagina, it is mainly composed of *Lactobacillus* [70]. We found that in FF, the microbiota from *Proteobacteria* dominated. This further confirms that our sample collection procedure minimizes contamination by vaginal and intestinal microbiota to the greatest extent possible.

PCOS‐associated microbial dysbiosis may reflect both pathogenic infiltration and procedural artifacts [71, 72]. In addition, there is endogenous microbial colonization in FF, which may arise through hematogenous dissemination (e.g., *Methylobacterium*) and selection of the follicular microenvironment. The presence of *Methylobacterium*, a genus occasionally isolated from bacteremia, and its inverse correlation with progesterone levels mirrors the gut–follicle axis hypothesis in patients with PCOS [73]. Furthermore, high D‐glucose in FF selectively enriches glucose‐utilizing *Firmicutes* (e.g., Gp4), whereas progesterone deficiency in individuals with PCOS may suppress *Lactobacillus* growth by limiting their metabolic substrates [74]. These mechanisms highlight how host physiology shapes the composition of the follicular microbiota.

The connections between these bacteria and metabolites are exciting. To further explain our conclusions from a biological perspective, we selected a key metabolite and validated the biological interactions with bacteria. Glucose was selected due to the well‐known key roles in PCOS and the significant correlation with microorganisms. Furthermore, we confirmed through a simulated follicular fluid environment that *Alicyclobacillus acidoterrestris*, *Terrimonas ferruginea*, and *Terrimonas pekingense* can metabolize glucose in follicular fluid [50, 75], providing biological evidence for the association between bacteria and metabolites, thereby achieving regulatory effects on PCOS. This provides insights into the bacteria–metabolite–PCOS association and biologically confirms the bacteria–metabolite correlation observed in follicular fluid. Of course, this is just an interesting example. Future investigations will focus on elucidating the functional roles of these pivotal microorganisms and metabolites one by one to comprehensively analyze their roles in the pathophysiology of PCOS.

A key strength of this study lies in the integrated multiomics approach used to study the microbiome and metabolome characteristics of FF, a comprehensive analysis that allowed us to assess microecological differences in FF more accurately in PCOS patients, providing mechanistic insights into microbial–metabolite crosstalk in PCOS associated ovulatory dysfunction. However, given the limitations of 16S rDNA sequencing technology, it is still challenging to identify the specific strains that are related to PCOS precisely, and this will be the focus of future research. The findings in this study broaden the understanding of this topic in related fields. The six strains screened in the study include numerous individual strains, each with distinct characteristics and may have different effects on follicle function and fertility. This study relied on the cross‐sectional design of a case–control study, which, while valuable for exploring associations between FF microbial composition, metabolite profiles, and ovarian function in PCOS patients at a single time point, inherently has its limitations. Specifically, it could not establish causal relationships between FF microecology and PCOS and may not capture dynamic microbial changes across variable clinical stages of PCOS progression. To address these limitations and advance our understanding of PCOS pathophysiology, future longitudinal studies are critical to track FF microecology during PCOS progression and integrate multiomics data to elucidate the microbial–host interactions that drive disease progression. Furthermore, clinical intervention trials (e.g., dietary modulation and probiotic supplementation) are needed to evaluate whether altering FF microecology can attenuate PCOS progression. Another limitation of this study is the inability to use specific antibiotics to eliminate specific bacterial genera, thereby validating their key role in the development of PCOS; thus, this suggests a new direction for future research.

## 4 Conclusion

In conclusion, our study highlights the significance of FF microecological dynamics as potential indicators or predictors of oocyte quality, and future studies should focus on identifying specific metabolites that may contribute to PCOS and further assessing the effects of abnormal bacterial genera on oocyte quality. Undoubtedly, elucidating the microecological composition of FF is critical for understanding the etiology of PCOS, gaining deeper insights, and ultimately improving its diagnosis and management. This study provides a foundation for further exploration of FF microecology in patients with PCOS, but much more work is needed to elucidate the complex relationships and mechanisms involved.

## 5 Materials and Methods

### 5.1 Patients

Forty patients with PCOS and 40 patients in the control group who underwent in vitro fertilization (IVF) or intracytoplasmic sperm injection (ICSI) at the Reproductive Center of Peking University Third Hospital were enrolled in this study from June 2022 to August 2022. The inclusion criteria were as follows: (1) women of reproductive age (20–40 years old); (2) patients with PCOS were enrolled if they met the following three features, including oligo‐ or anovulation and clinical/biochemical hyperandrogenism (defined in our center as androstenedione > 11.5 nmol/L, testosterone > 2.53 nmol/L, or dehydroepiandrosterone sulfate level > 10.6 µmol/L), and polycystic ovarian morphology (≥ 12 follicles per ovary), with the exclusion of other etiologies (congenital adrenal hyperplasia, androgen‐secreting tumors, Cushing's syndrome, etc.) [76], while women in the control group were required to have regular menstrual cycles, normal sex hormone levels, and normal ovarian morphology; (3) underwent a standard controlled ovarian stimulation protocol; and (4) complete clinical and IVF cycle‐related information. None of the enrolled patients received antibiotics or antimicrobial therapy during their IVF or ICSI cycles and had Grade I–II vaginal cleanliness. The results of the hydrogen peroxide and leukocyte esterase tests were negative. The study protocol was reviewed and approved by the Ethics Committee of Medical Scientific Research at Peking University Third Hospital. Written informed consent was signed by all participants (Peking University Third Hospital Ethics Committee No. 2021SZ‐011).

### 5.2 Assessment of Clinical Pregnancy Outcomes

Based on their specific clinical characteristics, patients underwent individualized stimulation protocols recommended by the hospital. Subsequently, oocyte retrieval was performed 36 h following the administration of human chorionic gonadotropin (hCG). Different fertilization methods were chosen by the patients after consulting with an experienced doctor. For IVF procedures, harvested oocytes were coincubated with 10,000 motile spermatozoa. For the ICSI group, mature oocytes (metaphase II [MII]) was confirmed by the extrusion of the first polar body (PB) prior to the procedure. Normal fertilization was identified by observing two pronuclei (2PN) and a second PB at 16–18 h after insemination. To determine the normal fertilization rate, the number of 2PN oocytes was divided by the total oocyte count in the IVF group, and by the MII oocyte count in the ICSI group. Embryo quality was evaluated using cell counts and fragmentation levels on Day 3. The transplantable embryo rate was defined as the ratio of eligible Day 3 embryos (≥ 5 cells and < 30% fragmentation) to the number of Day 2 cleaved embryos. Final embryo transfer occurred on Day 3, 5, or 6, depending on individual clinical circumstances.

The definitions for clinical outcomes were as follows: clinical pregnancy was confirmed by the detection of a gestational sac via transvaginal ultrasonography; live birth referred to the delivery of at least one living infant; and pregnancy complications mainly including gestational hypertension and gestational diabetes mellitus; preterm birth: delivery occurs before 37 weeks but 28 weeks of pregnancy; and low birth weight infants: newborns with a birth weight of less than 2500 g. Information regarding patient characteristics and clinical pregnancy outcomes was retrieved from the electronic medical records, which undergo regular quality assurance by a designated team.

### 5.3 Collection of Follicular Fluid

Each patient received an individualized stimulation protocol. Gonadotropin was injected daily beginning on Day 2 of the menstrual cycle, and the GnRH agonist was administered daily beginning on Day 5. Follicle size was monitored by transvaginal ultrasound and when at least three follicles were larger than 17 mm in diameter, the patient received an injection of GnRH‐a alone or combined with hCG. Thirty‐six hours after administration of the trigger injection, ovum pick‐up was performed under ultrasound guidance, and different fertilization methods were performed according to the protocol selected by the patient after consultation with an experienced doctor. All oocyte collection was carried out under strict aseptic conditions: the vulva and vagina were thoroughly disinfected before the operation, and the entire process was guided by transvaginal ultrasound. Sterile puncture needles and sterile gloves were used to ensure the safety and standardization of the operation.

All experiments were conducted under the same conditions, and nucleic acid‐free water without DNA samples was used as the negative control for subsequent microbial testing. FF from dominant follicles containing oocytes (diameter > 1.4 cm) were collected during ovum pick‐up. To avoid blood contamination of the sample, we used only the first tube of FF. The extracted FF was divided into equal parts, and one portion was collected in disposable sterile cryotubes for subsequent 16S rDNA sequencing analysis. The sterile tubes were stored at −20°C. The remaining FF sample was centrifuged at 3000 rpm for 10 min to remove granulosa cells. The centrifuged supernatant was collected and stored at −80°C until metabolomic analysis using liquid chromatography–tandem mass spectrometry (LC–MS/MS).

### 5.4 Metabolite Extraction and LC–MS/MS Analysis

The follicular fluid sample was slowly thawed at 4°C. In total, 100 µL of FF was added to a 1.5 mL centrifuge tube, 150 µL of prechilled 80% methanol was added, and the mixture was vortexed well for 10 min. The mixture was left on ice for 10 min and then centrifuged at 13,500 rpm for 20 min at 4°C. The supernatant fractions were carefully collected and subsequently evaporated using a vacuum concentrator. The resulting dried extracts were then reconstituted in 100 µL of a methanol/water mixture (1:1, v/v). After the reconstituted samples were vortexed for 30 s, they were centrifuged at 15,000 × *g* for 20 min at 4°C to eliminate any insoluble particles. The supernatants were aliquoted into LC–MS/MS vials preloaded with internal standards prior to analysis.

#### 5.4.1 Analytical Instrumentation

UPLC‐QTRAP 6500+: A Shimadzu ultrahigh‐performance liquid chromatography (UHPLC) system (Shimadzu Corporation, Japan) equipped with a binary solvent delivery system and an autosampler was used. The system was coupled to an AB SCIEX QTRAP 6500+ triple quadrupole linear ion trap mass spectrometer (PerkinElmer, USA) with an electrospray ionization (ESI) source.

#### 5.4.2 Chromatographic Conditions

A UPLC BEH C18 column (100 mm × 2.1 mm, 1.7 µm) was used with a column temperature of 45°C. Mobile phase A consisted of water (0.1% formic acid), and mobile phase B consisted of acetonitrile (0.1% formic acid). The flow rate was 0.3 mL/min, and the injection volume was 5 µL. Gradient elution was performed by initially holding phase B at 5% (0–1 min). Subsequently, B was increased linearly: 5%–25% (1–5 min), 25%–40% (5–15.5 min), and 40%–95% (15.5–17.5 min). After holding at 95% (17.5–19 min), the concentration returned to 5% (19–19.5 min), and was maintained for equilibration (19.6–21 min).

#### 5.4.3 Mass Spectrometric Conditions

ESI was performed in both positive (ESI+) and negative (ESI−) ion modes. The key parameters were as follows: curtain gas, 35 psi; ion source gas, 50 psi; auxiliary heating gas, 50 psi; collision gas medium; and ion source temperature, 550°C. For ESI+, the ionization voltage was set to 5500 V, whereas for ESI−, it was −4500 V. Data acquisition was performed in multiple reaction monitoring (MRM) mode.

#### 5.4.4 Determination of Target Diagnostic Marker Concentrations

Calibration curves were constructed by plotting the ratio of the peak area of the target diagnostic marker to that of its corresponding stable isotope‐labeled internal standard against the concentration of the standard solution. Quantification was performed by means of isotope‐labeled internal standards. Quality control during sample analysis was ensured by spiking samples with isotope‐labeled internal standards.

### 5.5 Metabolomic Data Analysis of Follicular Fluid

Raw data from LC–MS/MS were processed using Analyst 1.6.3 (AB Sciex, USA). The processing workflow involved peak extraction, intra‐ and intergroup retention time correction, as well as background peak labeling and metabolite identification. Subsequently, the R package “mixOmics” was employed to conduct PCA and PLS‐DA. We applied univariate analysis (*t*‐test) to evaluate statistical significance (*p* value). FDR correction (Benjamini–Hochberg) was applied to all the metabolite *p* values.

The criteria for identifying differentially abundant metabolites were a projective variable importance (VIP) score greater than 1, a *Q* value less than 0.05, and a multiple change (FC) greater than or equal to 0.58 or less than or equal to 0.58. Afterward, ggplot2 was used to generate volcano maps that visually demonstrated the differentially abundant metabolites. We subsequently further investigated metabolite‐related functions and metabolic pathways with the KEGG database.

### 5.6 DNA Preparation, PCR Amplification, and Sequencing

For bacterial DNA isolation, samples were initially homogenized using a Stomacher‐400 blender. DNA extraction was carried out with the QIAamp Stool Mini Kit (Qiagen, Venlo, the Netherlands). The manufacturer's protocol was modified by incubating samples with lysis buffer at 95°C rather than 70°C to enhance the lysis efficiency for both gram‐negative and gram‐positive bacteria. DNA concentration was measured using a NanoDrop ND‐1000 spectrophotometer (Thermo Fisher Scientific, DE, USA). The DNA yield was quantified based on absorbance at 260 nm, while purity was evaluated by determining the *A*_260_/*A*_280_ ratio (commonly accepted as ∼1.8 for pure DNA) to detect protein contamination, and the *A*_260_/*A*_230_ ratio (ideally > 1.5) to monitor residual salts or phenol.

To profile the microbiome, we sequenced the V3–V4 hypervariable regions of the 16S rRNA gene using an Illumina MiSeq system. The specific primers used were forward 5′‐CCTACGGGNGGCWGCA‐3′ and reverse 5′‐GACTACHVGGGTATCTAATCC‐3′. PCR amplification was carried out in a total volume of 25 µL, comprising 10 ng of extracted DNA (2.5 µL), 5 µL of each primer (1 µM), and 12.5 µL of 2X KAPA HiFi Hotstart ready mix (KAPA Biosystems, Woburn, MA, USA). Postamplification purification was achieved with AMPure XP beads (Beckman Coulter, Indianapolis, IN, USA). Subsequently, indexing PCR was performed using the Nextera XT Index Kit (Illumina, San Diego, CA, USA). The amplified products were pooled and again purified with AMPure XP beads (Beckman Coulter) prior to quantification. Final libraries underwent paired‐end sequencing (2 × 300 bp) on the Illumina MiSeq system with a MiSeq Reagent Kit v.3.

### 5.7 Quality Control and Analysis of 16S rDNA Sequencing Data

First, the original sequence data were processed to remove low‐quality sequences and fit sequences. Sequence analysis was carried out via Mothur (version 1.43.0) based on the MiSeq SOP, utilizing SILVA (version 138.1) data obtained from the Mothur site. After alignment and chimera removal, sequences sharing ≥ 97% similarity were clustered into operational taxonomic units (OTUs). In the Conet analysis, OTUs whose total sequences were greater than 0.02% were screened for further analysis.

PCoA analyses using the cmdscale function were used to examine β‐diversity to gain insight into the differences in the microbial communities between different populations. The phyloseq R package was used to obtain eight different metrics to quantify alpha diversity. PCA was used for cluster analysis. In this analysis, dimension reduction of the original variables was performed via the ade4 software package and ggplot2 software package in R software (version 4.3.1). Principal coordinate analysis (PCoA) was then used to visualize the differences in multidimensional data between different groups. In addition, a linear discriminant analysis effect size (LDA) analysis was performed using LEfSe software (version 1.0) to identify potential biomarkers specific to PCOS. LEfSe (version 1.0) was run with default parameters: a Kruskal–Wallis test followed by a pairwise Wilcoxon test, both with Benjamini–Hochberg FDR < 0.05; and an LDA score threshold = 4.0.

### 5.8 Construction of Predictive Models Through Machine Learning Methods

The included biomarkers were identified prior to difference analysis (*p* < 0.05 + FDR correction) rather than through data‐driven feature selection. Four algorithms, namely, BLR, DT, SVM, and RF, were adopted to construct prediction models based on the selected biomarkers. We included all 40 pairs of patients (*n* = 40 PCOS patients vs. *n* = 40 controls) and randomly divided the cohort into a 70% training set and a 30% test set. The pROC package was used to construct ROCs and evaluate the AUCs, which were used to measure the predictive performance in the training and test sets. The model constructed by the best algorithm was selected, and the efficacy of the model was further verified in an external validation set (*n* = 20 PCOS patients vs. *n* = 20 controls). The mean decrease in accuracy and mean decrease in the Gini coefficient were used to calculate the importance of the variables in the model, which were positively associated with the importance.

### 5.9 Bacterial Culture

Standard BHI medium was used for bacterial cultivation. Specifically, 100 µL of preactivated microorganisms was injected into 5 mL of sterile BHI medium and incubated at 37°C for 1 day. They were subsequently coincubated with glucose to evaluate their ability to metabolize substrates. For follicular fluid, 100 µL of preactivated microorganisms was injected into 1 mL of FF from patients with PCOS for incubation, after which the glucose and testosterone levels were measured 1 day later.

### 5.10 Statistical Analysis

SPSS Statistics (version 27.0; IBM Corp., Armonk, New York), Prism (version 10) and R software were utilized for data processing. All the charts were drawn using R, Prism (version 10), Adobe Illustrator (version 2025), and BioRender (https://biorender.com). Continuous variables were presented as either the median with interquartile range (IQR) for non‐normally distributed data, or the mean with standard deviation (SD) for normally distributed data. The normality of data was assessed using the Shapiro–Wilk test, as the sample size was limited. For variables exhibiting a normal distribution, comparisons were made using Student's *t*‐test (two groups) or one‐way ANOVA (multiple groups). Post hoc analyses for ANOVA included Tukey's test (the same SD) or Dunnett's T3 test (different SD). Conversely, non‐parametric data were analyzed via the Mann–Whitney *U*‐test (two groups) or the Kruskal–Wallis test (multiple groups). For categorical data, statistical significance was evaluated via chi‐square tests or Fisher's exact test. Using R software, we employed the psych package (version 2.4.3) for Spearman correlations and the pROC package (version 1.18.5) for the construction of ROCs and determination of AUCs. A two‐tailed *p* value of less than 0.05 was considered statistically significant.

## Author Contributions

Jie Qiao and Xinyu Qi had full access to all of the data in the study and take responsibility for the integrity of the data and the accuracy of the data analysis. *Concept and Design*: Jie Qiao, Xinyu Qi. *Acquisition, Analysis, or Interpretation of Data*: Manfei Si, Sen Yan, Shu Ding, Rui Liu, Xianglei Xiong. *Drafting of the Manuscript*: Manfei Si, Shu Ding. *Critical Review of the Manuscript for Important Intellectual Content*: Manfei Si, Sen Yan, Shu Ding, Rui Liu, Xianglei Xiong, Jie Qiao, Xinyu Qi. *Statistical Analysis*: Manfei Si, Sen Yan, Shu Ding. *Obtained Funding*: Manfei Si, Sen Yan, Jie Qiao, Xinyu Qi. Supervision: Xinyu Qi, Jie Qiao. All the authors read and approved the final manuscript.

## Funding

This work was supported by the National Key Research of Development Program of China 2024YFC2707300, the National Natural Science Foundation of the Peoples’ Republic of China (82202894, 82301841), the Beijing Research Ward Excellence Program (BRWEP2024W0940901004), the Beijing Natural Science Foundation 7242162, the Key Clinical Projects of Peking University Third Hospital (No. BYSY2022045), and the Joint Research Project of the Shijiazhuang‐Peking University Cooperation Program.

## Ethics Statement

The protocol for this study was approved by the Ethics Committee of Peking University Third Hospital (approval No. 2021SZ‐011).

## Consent

Written informed consents were obtained from participants for research purposes prior to the study in accordance with the Declaration of Helsinki.

## Conflicts of Interest

The authors declare no conflicts of interest.

## Supporting information




**Table S1** IVF and clinical pregnancy outcomes in the PCOS and control groups.
**Figure S1** (A) PCA revealed discernible disparities in metabolite distribution between the control and PCOS groups. (B) The alpha diversity analysis revealed the species diversity of the PCOS group was lower than that of the control group. PCA, principal component analysis.

## Data Availability

The raw sequence data in this study have been deposited in the database of the National Center for Biotechnology Information (https://www.ncbi.nlm.nih.gov/) under accession number PRJNA1363685, and the data are available from the corresponding author upon request.
