# Supplementary material for: Changes in Metabolites and Microbial Communities in Follicular Fluid Associated With Ovarian Function in Patients With Polycystic Ovary Syndrome
Source: MedComm (2020). 2026 Jan 29;7(2):e70622. doi: 10.1002/mco2.70622 (PMC12856059; doi:10.1002/mco2.70622)
Supplement: Supplementary file 1 — Table S1 IVF and clinical pregnancy outcomes in the PCOS and control groups. Figure S1 (A) PCA revealed discernible disparities in metabolite distribution between the control and PCOS groups. (B) The alpha diversity analysis revealed the species diversity of the PCOS group was lower than that of the control group. PCA, principal component analysis. [file MCO2-7-e70622-s001.docx]

**Changes in metabolites and microbial communities in follicular fluid associated with ovarian function in patients with polycystic ovary syndrome**

**Running Head**

Metabolite and microbiome changes in FF of PCOS

Manfei Si^1,2,3,4,#^, Sen Yan^1,2,3,4,#^, Shu Ding^1,2,3,4,#^, Rui Liu^5^, Xianglei Xiong^1,2,3,4^, Jie Qiao^1,2,3,4,*^, Xinyu Qi^1,2,3,4,*^

^1^ State Key Laboratory of Female Fertility Promotion, Center for Reproductive Medicine, Department of Obstetrics and Gynecology, Peking University Third Hospital, Beijing, China

^2^ National Clinical Research Center for Obstetrics and Gynecology (Peking University Third Hospital), Beijing, China

^3^ Key Laboratory of Assisted Reproduction (Peking University), Ministry of Education, Beijing, China

^4^ Beijing Key Laboratory of Reproductive Endocrinology and Assisted Reproductive Technology, Beijing, China

^5^ Institute of Advanced Clinical Medicine, Peking University, Beijing, China

^#^ Manfei Si, Sen Yan and Shu Ding are contributed equally.

***Corresponding Author:** Xinyu Qi, PhD, and Jie Qiao, PhD, Center for Reproductive Medicine, Department of Obstetrics and Gynecology, Peking University Third Hospital, No. 49 North Huayuan Road, Haidian District, Beijing 100191, China ([qixinyu@bjmu.edu.cn](mailto:qixinyu@bjmu.edu.cn) and jie.qiao@263.net).

**Supplementary Table 1.** IVF and clinical pregnancy outcomes in the PCOS and control groups.

| **Variable** |  | **PCOS**  **(n = 40)** | **Control**  **(n = 40)** | ***P* value** |
| --- | --- | --- | --- | --- |
| IVF outcomes | Normal fertilization rate* | 0.71 (0.32) | 0.71 (0.28) | 0.606 |
|  | Cleavage rate* | 1.00 (0.00) | 1.00 (0.00) | 0.569 |
|  | High quality embryo rate* | 0.78 (0.36) | 0.90 (0.33) | 0.277 |
|  | Available embryo rate* | 0.62 (0.31) | 0.50 (0.33) | 0.201 |
|  | Transfer strategy |  |  |  |
|  | Fresh-Embryo Transfer | 8/37 (21.6%) | 21/35 (60.0%) | 9.05E-4 |
|  | Frozen-Embryo Transfer | 29/37 (78.4%) | 14/35 (40.0%) |  |
| First transplantation | Clinical pregnancy | 22/37 (59.5%) | 18/35 (51.4%) | 0.493 |
|  | Live birth | 16/22 (72.7%) | 16/18 (88.9%) | 0.258 |
|  | Pregnancy complications | 2/16 (12.5%) | 2/16 (12.5%) | 1.000 |
|  | Preterm birth | 2/16 (12.5%) | 4/16 (25.0%) | 0.654 |
|  | Low birth weight infants | 1/16 (6.3%) | 3/16 (18.8%) | 0.600 |
| Cumulative transplantation | Clinical pregnancy | 31/37 (83.8%) | 25/35 (71.4%) | 0.208 |
|  | Live birth | 26/31 (83.9%) | 22/25 (88.0%) | 0.720 |
|  | Pregnancy complications | 3/26 (11.5%) | 3/22 (13.6%) | 1.000 |
|  | Preterm birth | 5/26 (19.2%) | 5/22 (22.7%) | 1.000 |
|  | Low birth weight infants | 4/26 (15.4%) | 3/22 (13.6%) | 1.000 |

*Note:* Continuous data were reported as the mean (standard deviation) for normally distributed data or the median (interquartile range) for non-normally distributed data*. Categorical data were reported as n (%). The t test or Mann‒Whitney U test was used to analyze continuous data, and the chi‒square test or Fisher’s exact test was used to analyze categorical data.

Abbreviations: IVF, in vitro fertilization.

**Figure S1.** (A) PCA revealed discernible disparities in metabolite distribution between the control and PCOS groups. (B) The alpha diversity analysis revealed the species diversity of the PCOS group was lower than that of the control group.

Abbreviations: PCA, principal component analysis.
